# Supplementary material for: The structural basis for the phospholipid remodeling by lysophosphatidylcholine acyltransferase 3
Source: Nat Commun. 2021 Nov 25;12:6869. doi: 10.1038/s41467-021-27244-1 (PMC8617236; doi:10.1038/s41467-021-27244-1)
Supplement: Supplementary file 1 — Supplementary Information [file 41467_2021_27244_MOESM1_ESM.pdf]

Supplementary information for

The structural basis for the phospholipid remodeling by lysophosphatidylcholine acyltransferase 3

Qing Zhang[1†], Deqiang Yao[2,3†], Bing Rao[2,4†], Liyan Jian[2,4], Yang Chen[2], Kexin Hu[2], Ying Xia[2,4], Shaobai Li[2], Yafeng Shen[2], An Qin[4], Jie Zhao[4], Lu Zhou[5], Ming Lei[2], Xian-Cheng Jiang[6], Yu Cao[2,4*]

Supplementary items:
Supplementary Figures 1-11
Supplementary Tables 1-3

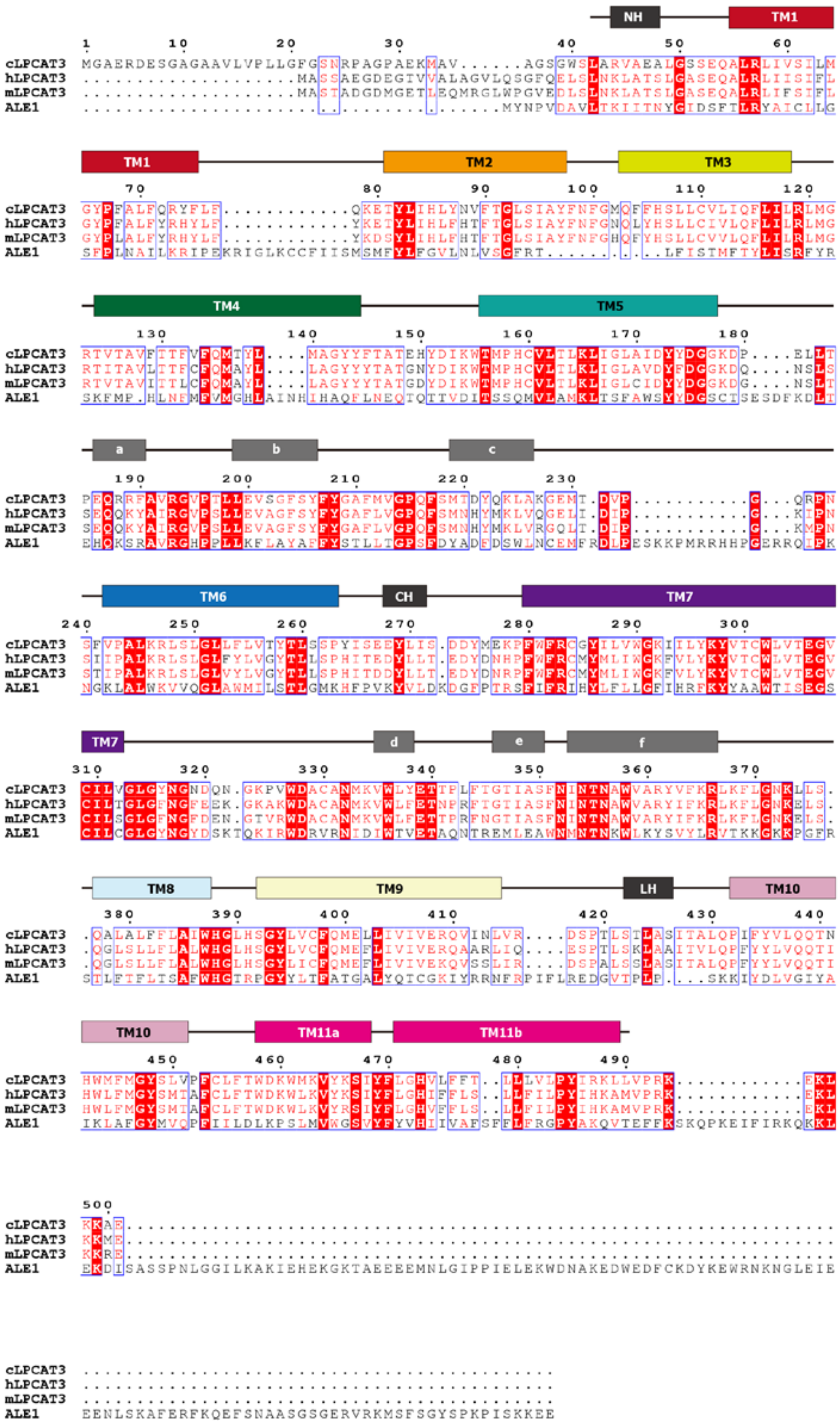

Supplementary Figure 1. Amino acid sequence alignment among LPCAT3 from human, mouse, chicken and yeast. The protein sequences of LPCAT3 from *Gallus gallus* (cLPCAT3, UniProtKB - A0A1L1RNG8, https://www.uniprot.org/uniprot/A0A1L1RNG8), *Homo sapiens* (hLPCAT3, UniProtKB - Q6P1A2, https://www.uniprot.org/uniprot/Q6P1A2), *Mus musculus* (mLPCAT3, UniProtKB - Q9DA37, https://www.uniprot.org/uniprot/Q9DA37) and *Saccharomyces cerevisiae* (ALE1, UniProtKB - Q08548, https://www.uniprot.org/uniprot/Q08548) were aligned

with the secondary structural elements of cLPCAT3 marked above the alignment. Residues are colored based on their conservation using the ESPript server.

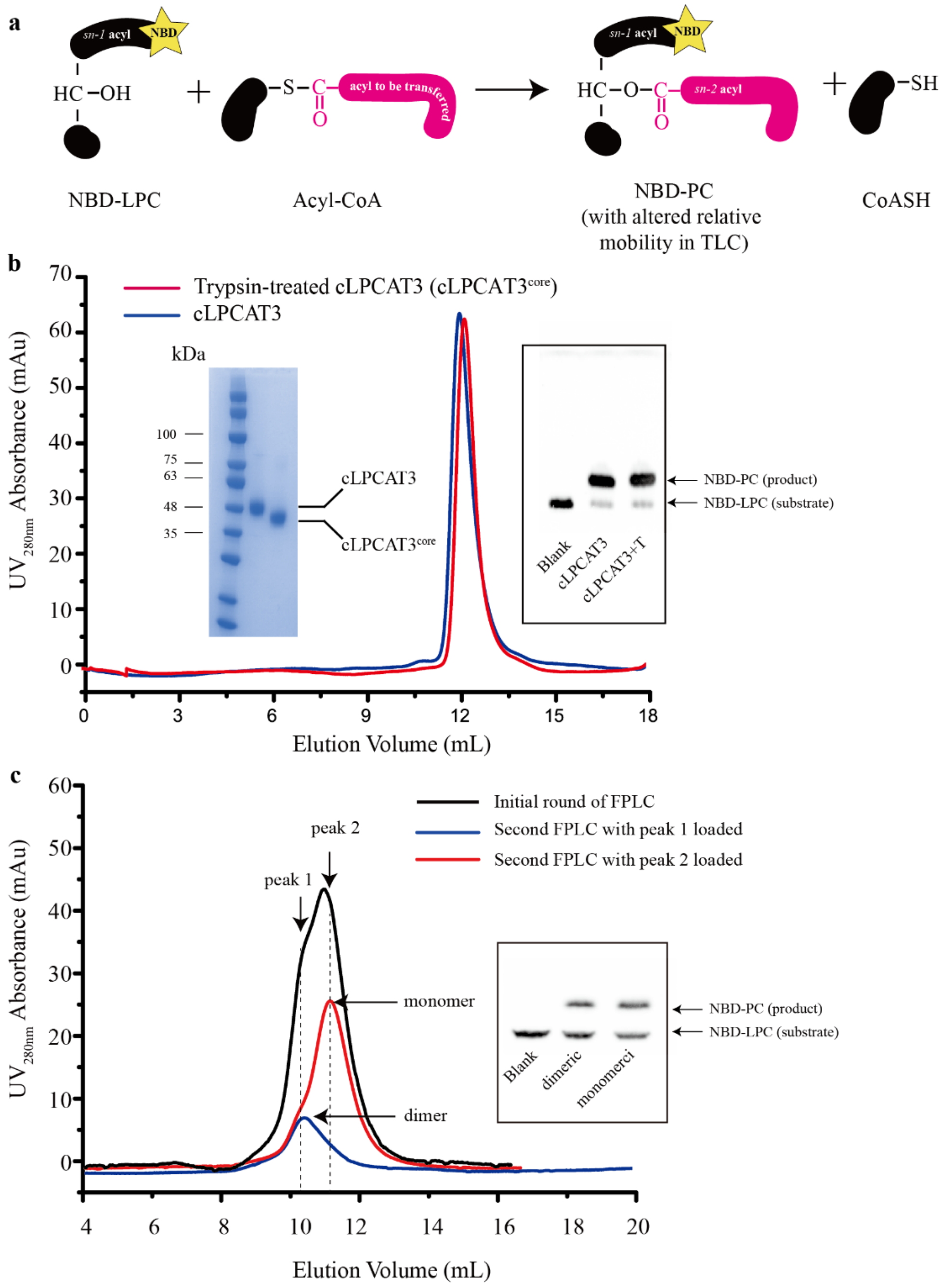

Supplementary Figure 2. The purification of the cLPCAT3^{core} for x-ray crystallography and the cLPCAT3 for cryo-electron microscopy. (a) the schematic diagram for the fluorescence-based measurement of LPCAT3 activity. (b) the size-exclusion chromatography profiles of cLPCAT3 and trypsin-digested cLPCAT3 (cLPCAT3^{core}). The proteins was solubilized with DDM and subjected to a Superdex 200 Increase 10/300 GL column in a mobile phase containing 150 mM NaCl, 20 mM HEPES, pH 7.5, 1 mM TCEP, and 1.2 mM UM. The experiment has been repeated three times with similar results. (c): the separation of dimeric and monomeric cLPCAT3. The cLPCAT3 eluate from affinity chromatography was subjected to size-exclusion chromatography (black curve). The fractions corresponding to peak 1 and 2 were pooled separately and subjected to further size-exclusion chromatography, respectively (red curve: further size-exclusion chromatography profile for peak 1; blue curve: further size-exclusion chromatography profile for peak 2). The experiment has been repeated three times with similar results. Insets of (b) and (c): The TLC assay was used to

estimate the enzymatic activities for the fractions as indicated, and similar results were achieved during more than three times repeat.

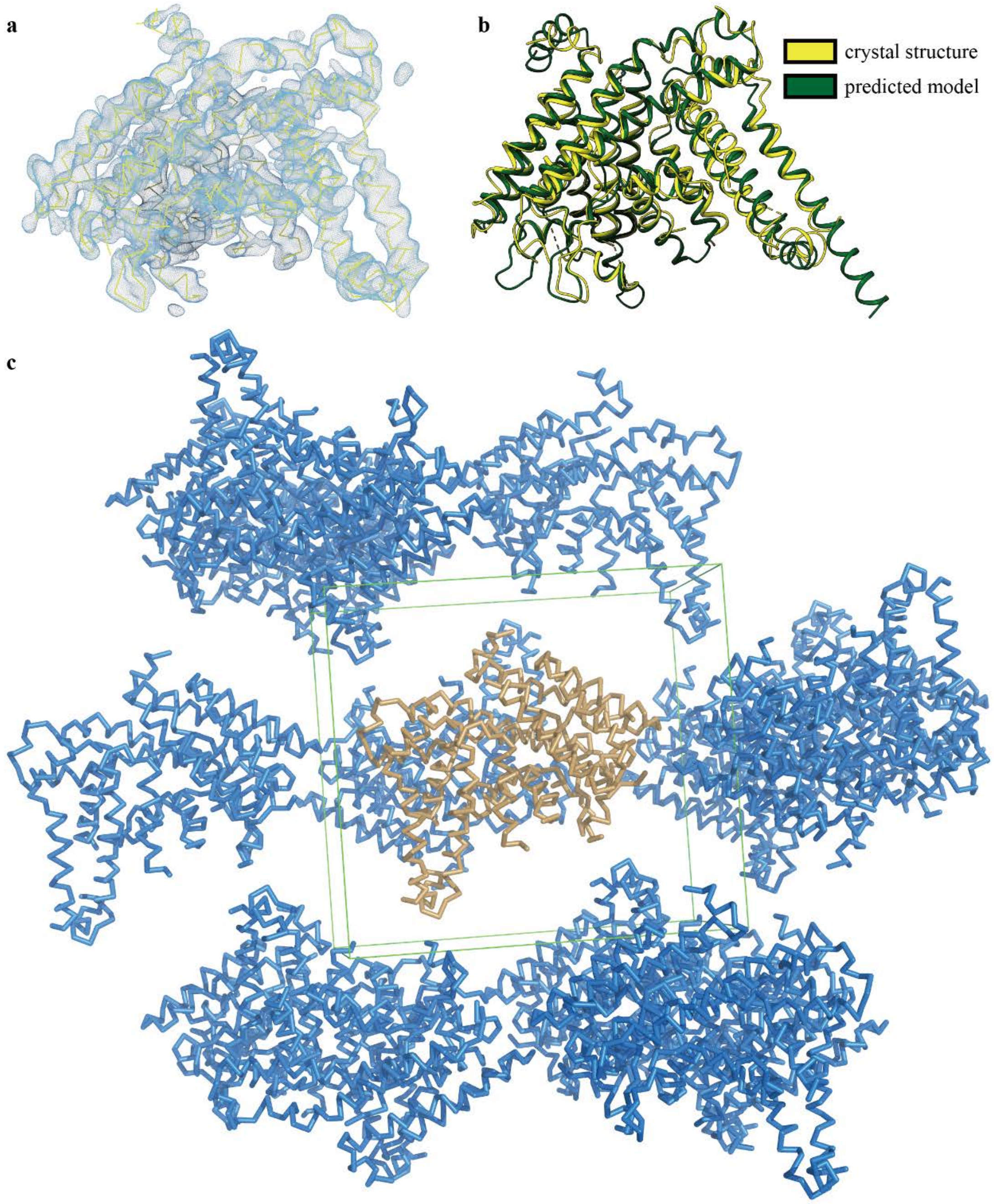

Supplementary Figure 3. The X-ray Crystallography of cLPCAT3.

(a) The molecular model of the crystal structure of cLPCAT3^{core} (yellow) and the corresponding electron density. The Fo-Fc electron density map was contoured to 1.5 σ. (b) The structural superposition between the crystal structure of cLPCAT^{core} and the molecular model predicted by Tencent tFold server (https://drug.ai.tencent.com/console/cn/tfold), which is used as the starting model in molecular replacement. The RMSD is about 2.094 Å. (c) The Crystal packing of cLPCAT3^{core}. The original molecular model to generate the crystal packing matrix was shown in yellow and others in blue.

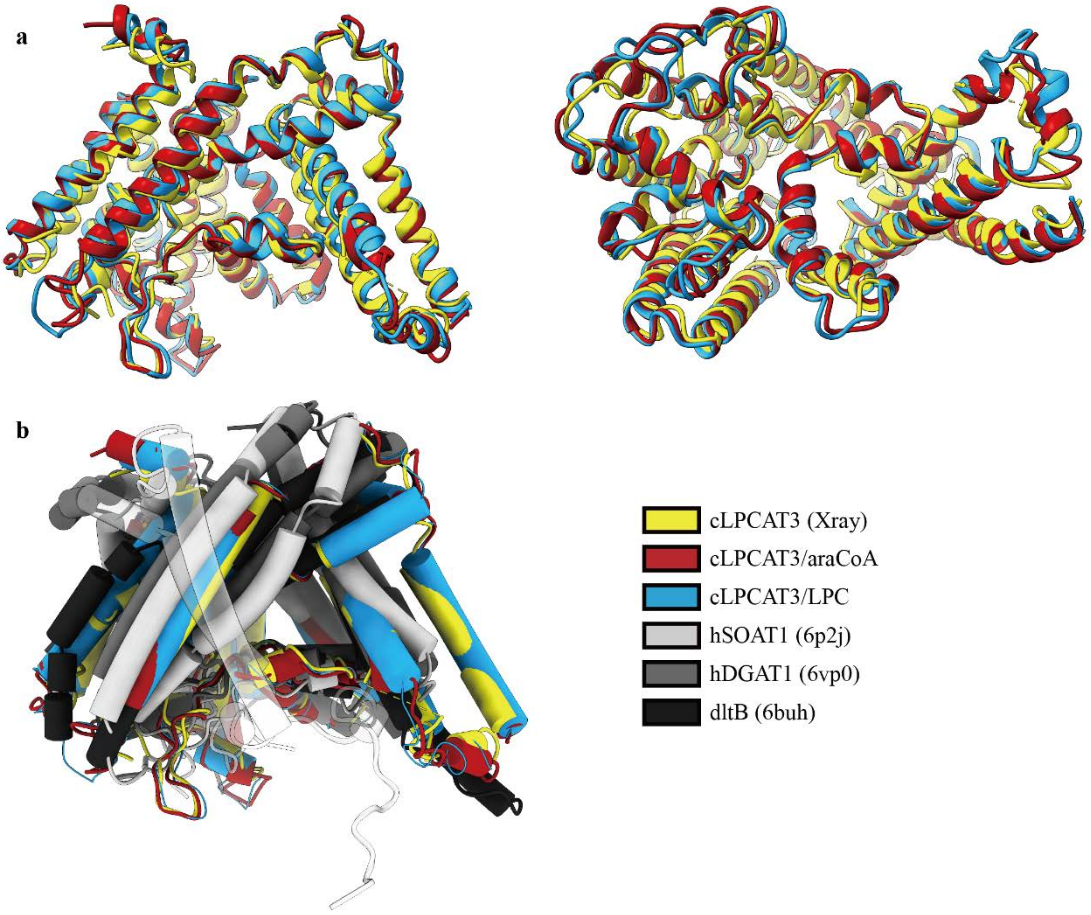

Supplementary Figure 4. The Structural superposition among the LPCAT3 in different states and other MBOAT members.

(a) The structural superposition among LPCAT3 protomers in apo (yellow), LPC-bound state (blue), and araCoA bound state (red). The Cartoon representation of the models were viewed parallel to the ER membrane (left) and from the lumen side of the ER membrane (right). (b) The structural superposition among the LPCAT3 protomers in different states with protomers from human DGAT1 (dark gray, PDB ID 6VP0), human SOAT1 (light gray, PDB ID 6p2j), and teichoic acid D-alanyltransferase (dltB) from *Streptococcus thermophilus* (black, PDB ID 6buh).

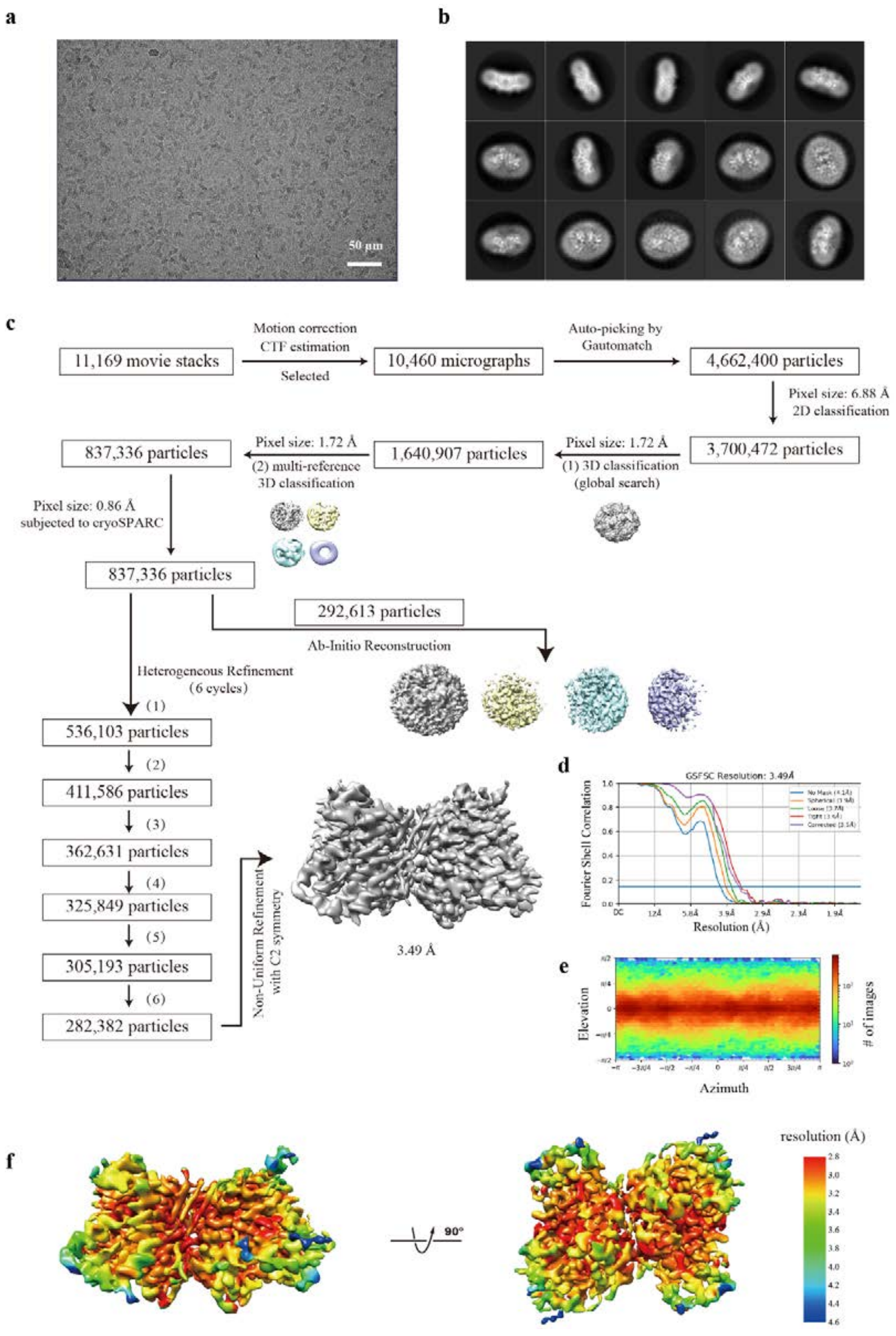

Supplementary Figure 5. The Cryo-EM analysis of cLPCAT3/araCoA. (a): A representative micrograph of cLPCAT3/araCoA. Most of the micrographs were similar with high quality. (b): A representative 2D class averages. (c): The flow chart of cryo-EM data processing on cLPCAT3/araCoA. (d): The gold-standard Fourier shell correlation (FSC) curve for the final cryo-EM map of cLPCAT3/araCoA, generated by cryoSPARC with non-uniform refinement. (e): Orientation distribution of particles for the final map reconstruction. (f): Local-resolution map shown in two orientations.

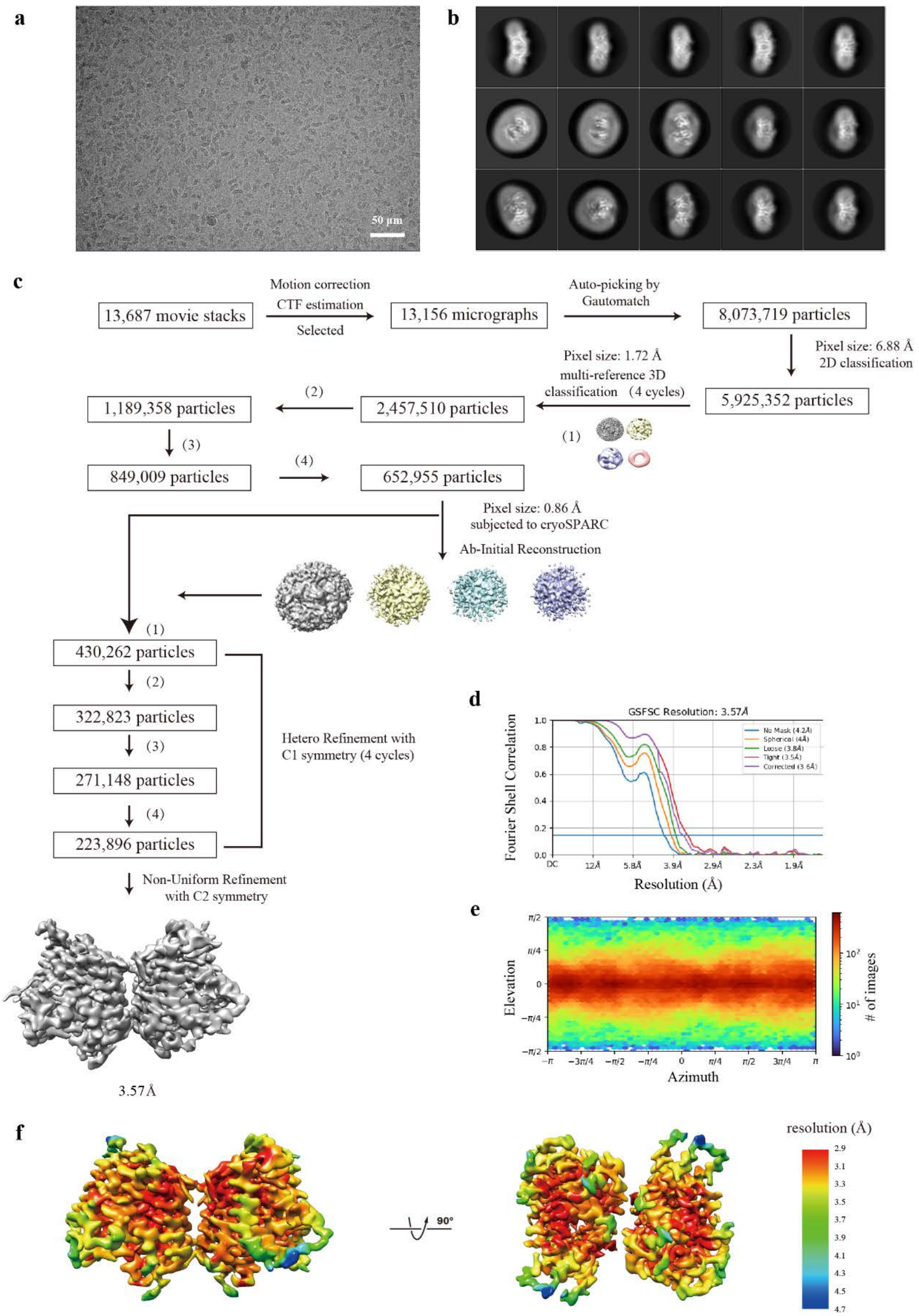

Supplementary Figure 6: Cryo-EM analysis of cLPCAT3/LPC. (a): A representative micrograph of cLPCAT3/LPC. Most of the micrographs were similar with high quality. (b): A representative 2D class averages. (c): The flow chart of cryo-EM data processing on cLPCAT3/LPC. (d): The gold-standard Fourier shell correlation (FSC) curve for the final cryo-EM map of cLPCAT3/LPC, generated by cryoSPARC with non-uniform refinement. (e): Orientation distribution of particles for the final map reconstruction. (f): Local-resolution map shown in two orientations.

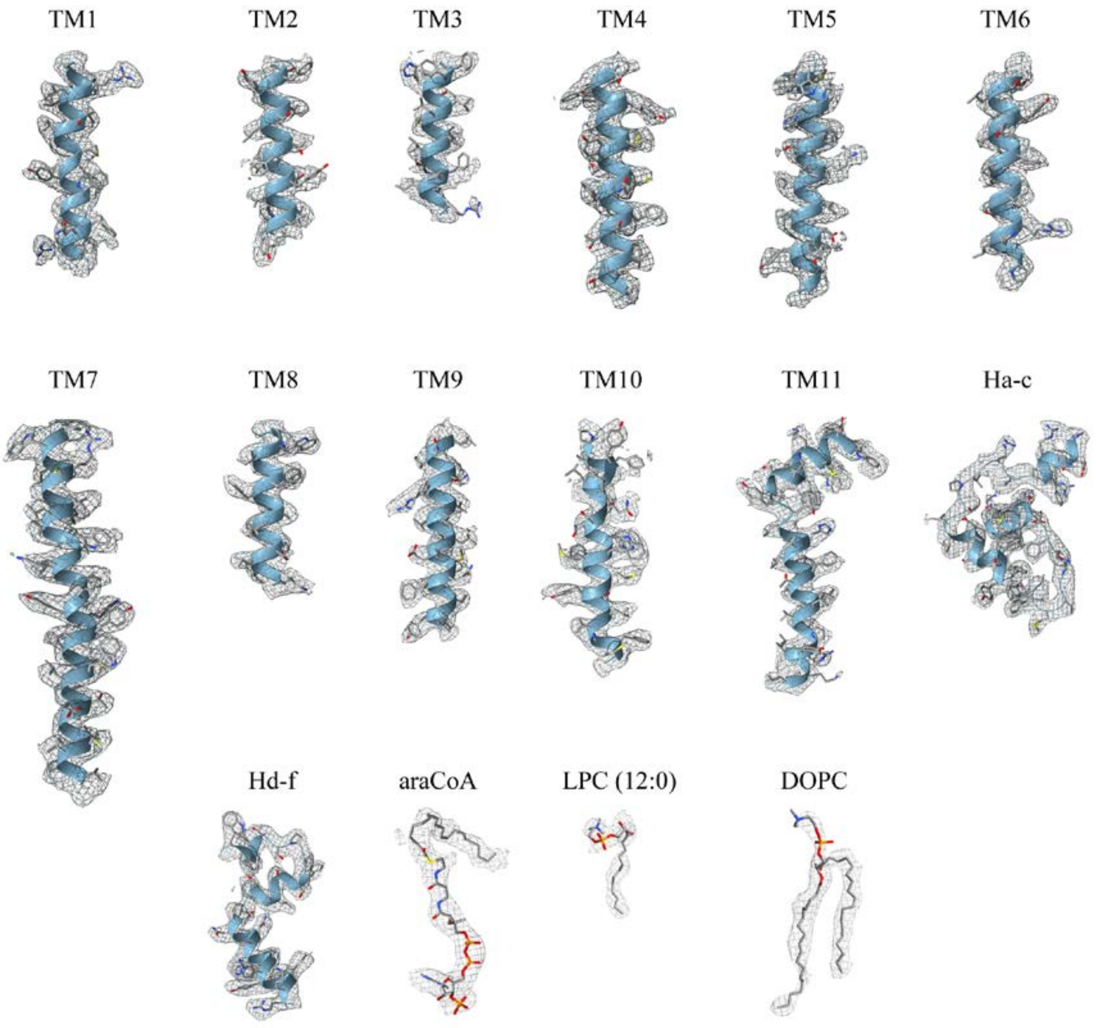

Supplementary Figure 7: The density and the model-fitting for the key secondary structures of cLPCAT3.

Individual transmembrane helices and Ha-f of cLPCAT3 were shown combined with their corresponding cryo-EM density map. The density maps for ara-CoA, LPC (12:0) and DOPC are shown at the same contour level as the protein structures.

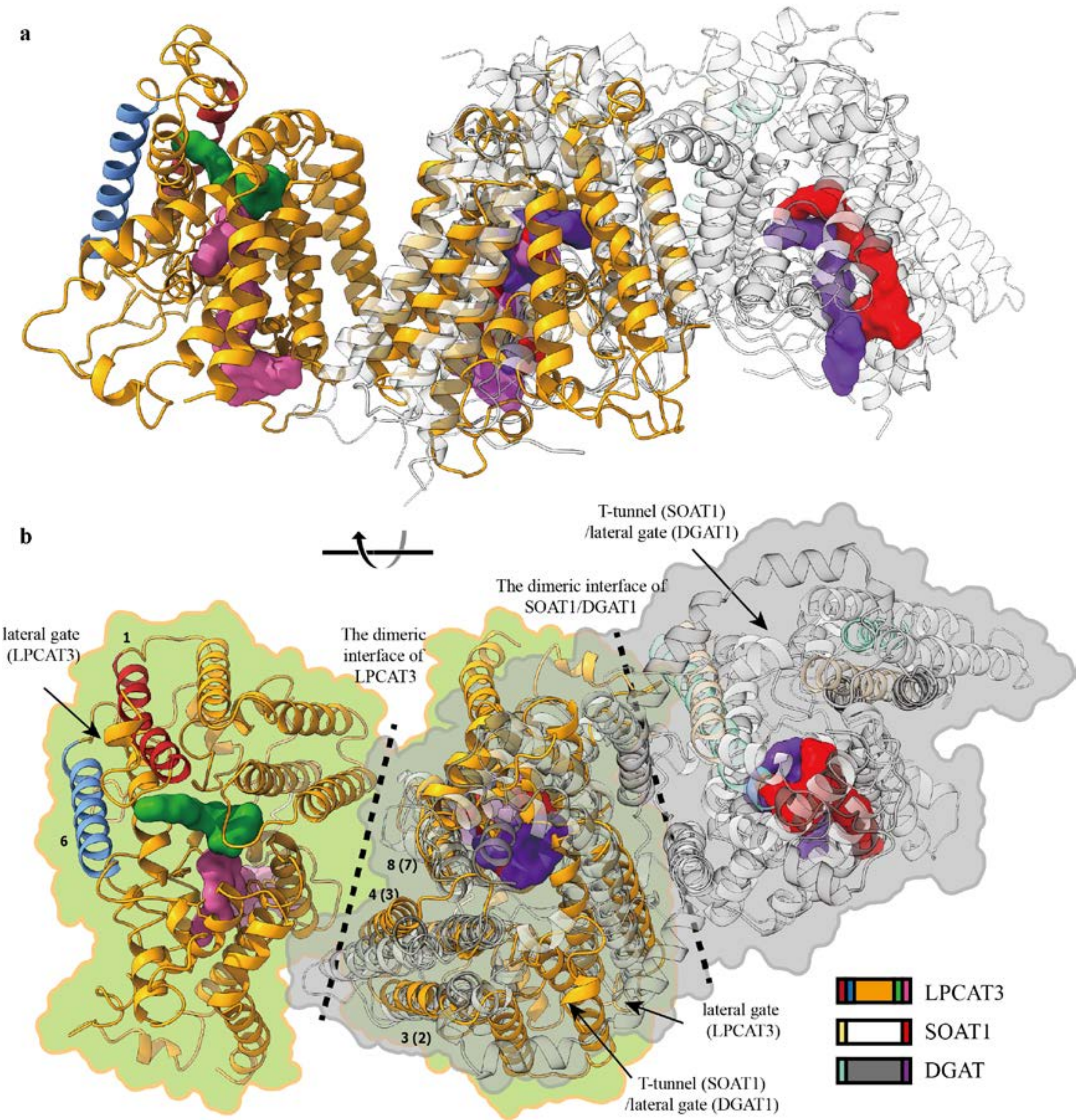

Supplementary Figure 8. A comparison among the dimers of MOBAT members. The structures of the dimeric cLPCAT3/araCoA, hSOAT1, and hDGAT1 were superposed using one cLPCAT3/araCoA promoter as the reference and the structures of the dimeric cLPCAT3/LPC was then superposed with cLPCAT3/araCoA to show the LPC molecule. The dimeric cLPCAT3/araCoA was highlighted with light green shading, and the dimeric hSOAT1 and hDGAT1 with gray shading. All protein structures for cLPCAT3/araCoA, hSOAT1, and hDGAT1 were shown as cartoon models, and the major bodies and highlighted helices were colored as the color palette shown at the lower right corner. The acyl-CoA molecules bound with enzymes, as well as the LPC molecule, were shown as the surface models and colored as the color palette. The cLPCAT3 helices TM3, 4, and 8 at the dimeric interface were labeled with the corresponding helices in hSOAT1 and hDGAT1 shown in parentheses. The structural models for hSOAT1 and hDGAT1 were generated with coordinate files of PDB accession numbers 6p2j and 6pv0, respectively.

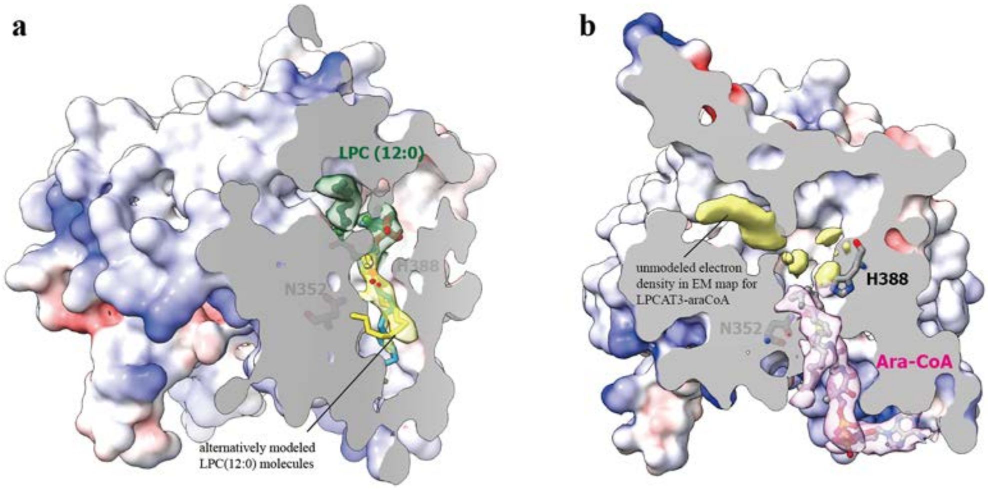

Supplementary Figure 9. **The unmodeled electron density in EM maps of cLPCAT3.**

(a) The surface model of cLPCAT3/LPC was dissected to show the LPC (12:0) molecules, LPC electron density (in green) and unmodeled density (in yellow). The LPC molecules in molecular model of cLPCAT3/LPC was shown as stick-and-ball model with the carbon atoms in green color. The LPC molecules in two alternative conformations were manually fit in the unmodeled density and shown as stick model with the carbon atoms in yellow and cyan color, respectively. (b) The surface model of cLPCAT3/araCoA was dissected to show the araCoA molecule, araCoA electron density (in pink) and unmodeled density (in yellow). The araCoA molecule was shown as stick-and-ball model with the carbon atoms in gray color.

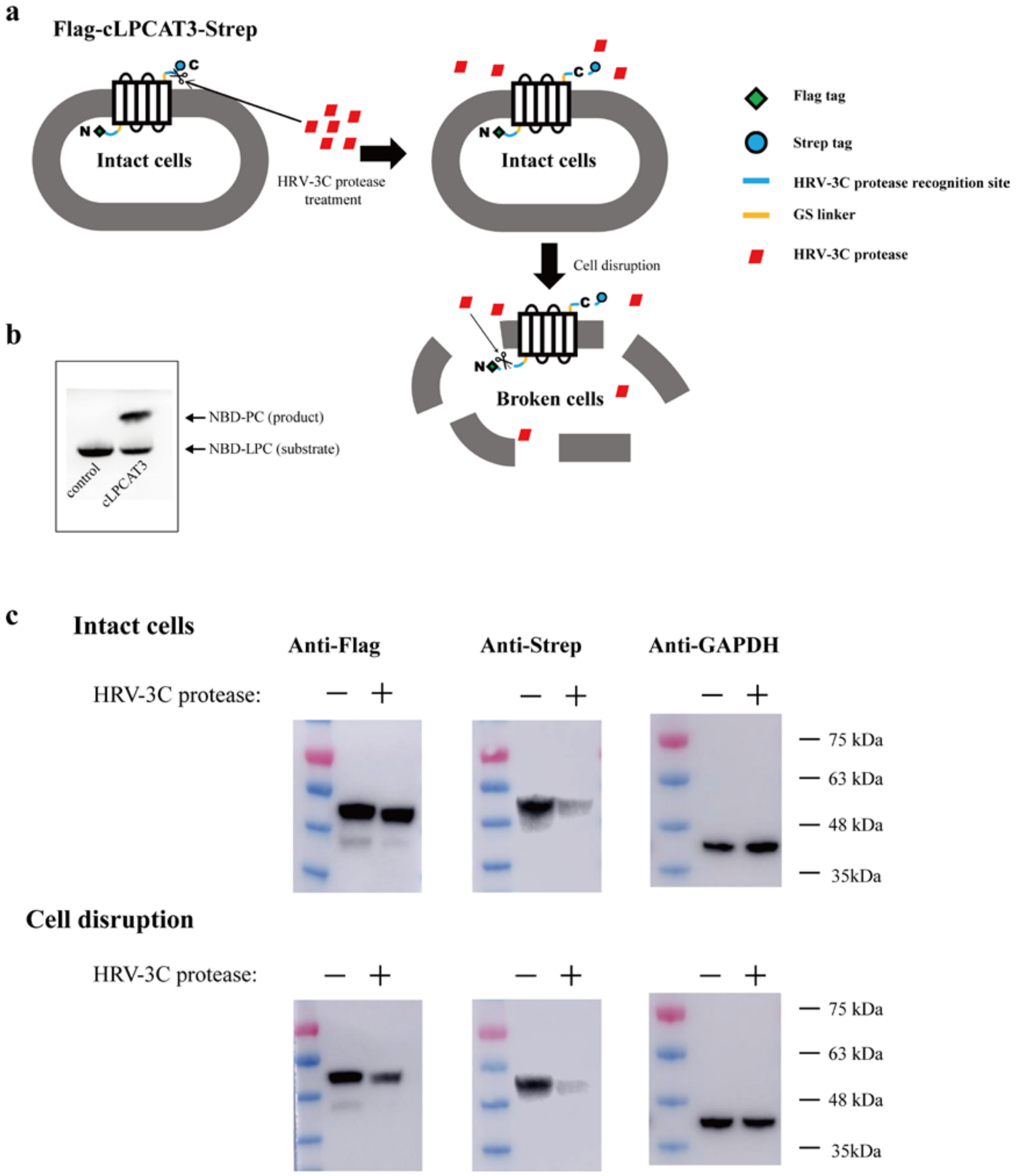

Supplementary Figure 10. On-cell protease digestion assay.

(a) The experimental principle for the on-cell protease digestion assay. The plasma membrane of the intact cells allow the HRV-3C protease to cut the tag exposing at exoplasmic environment but prevents it from cutting the tag within the cytoplasm, while the cleavage on both tags could start after the cell disruption. (b) the TLC assay on the enzymatic activities of purified Flag-cLPCAT3-strep proteins. The experiments were repeated three times with similar results. (c) The Western blot analysis of the on-cell cleavage efficiency of HRV-3C protease on Flag-cLPCAT3-Strep. The blotting band remain its intensity unchanged (left panel) for Flag tag, implying the cytoplasmic localization of the N-terminal of LPCAT3, while the blotting band for Strep tag reduced upon the digestion, implying the exoplasmic localization of the C-terminal of LPCAT3. The intensity of the blotting band for both tags reduced significantly when conducting the digestion after cell disruption. The experiments were repeated three times with similar results.

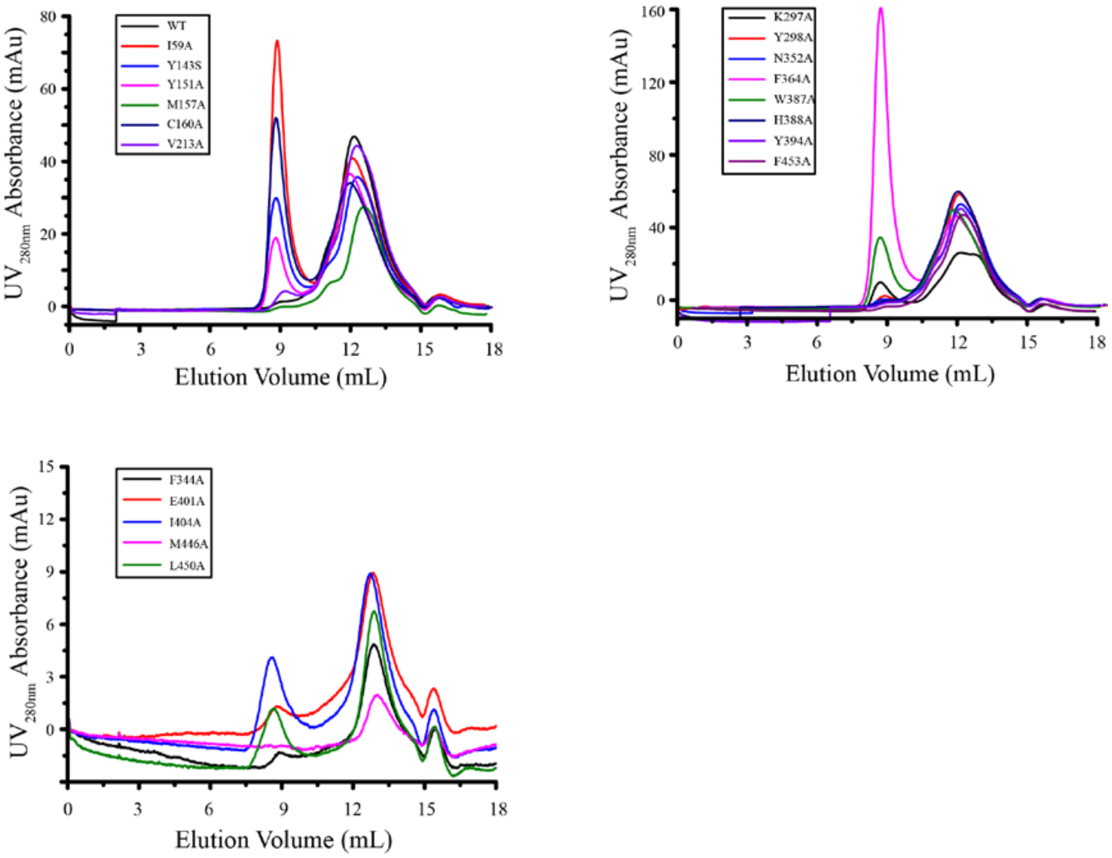

Supplementary Figure 11. The chromatography profiles for the gel filtration analysis of the cLPCAT3 mutants.

Supplementary Tables

Supplementary Table 1. The data collection and refinement statistics for cLPCAT3^{core} crystal structure.

	**Chicken PCAT3^{core} (PDB ID 7EWT)**
**Data collection**	
Space group	P 21 21 21
Cell dimensions	
a, *b*, *c* (Å)	76.79, 82.26, 116.92
α, β, γ (°)	90.00, 90.00, 90.00
Resolution (Å)	3.40
R_{sym} or R_{merge}	0.069 (1.892)
I / σI	21.84(1.33)
Completeness (%)	99.8(99.3)
Redundancy	12.9(12.9)
**Refinement**	
Resolution (Å)	3.40
No. reflections	10597
R_{work} / R_{free}	0.351 / 0.376
No. atoms	
Protein	3430
Ligand/ion	n/a
Water	n/a
B-factors	
Protein	158.01
Ligand/ion	n/a
Water	n/a
R.m.s. deviations	
Bond lengths (Å)	0.009
Bond angles (°)	1.02

Supplementary Table 2. Summary of cryo-EM data collection, processing and structure refinement.

	LPCAT3/araCoA (EMDB-31443) (PDB 7F40)	LPCAT3/LPC (12:0) (EMDB-31442) (PDB 7F3X
**Data collection and processing**		
Magnification	105,000	105,000
Voltage (kV)	300	300
Electron exposure (e-/Å^2)	50	50
Defocus range (μm)	-1.0 to -2.5	-1.0 to -2.5
Pixel size (Å/pixel)	0.86	0.86
Symmetry imposed	C2	C2
Initial particle images (no.)	4,662,400	8,073,719
Final particle images (no.)	282,382	223,689
Map resolution (Å)	3.49	3.57
FSC threshold	0.143	0.143
Map resolution range (Å)	2.8-4.6	2.9-4.7
**Refinement**		
Initial model used (PDB code)	7EWT	7EWT
Model resolution (Å)	3.9	3.9
FSC threshold	0.5	0.5
Map sharpening B factor (Å^2)	-184	-174
Model composition		
Non-hydrogen atoms	7488	7408
Protein residues	898	898
Ligands	AAC: 2	LYC: 2
B factors (Å^2)		
Protein	47.88	88.52
Ligand	35.69	71.22
R.m.s. deviations		
Bond length (Å)	0.003	0.003
Bond angles (°)	0.774	0.638
Validation		
MolProbity score	1.91	1.8
Clashscore	7.64	6.07
Rotamer outliers (%)	0.51	0.00
Ramachandran plot		
Favored (%)	91.72	92.62
Allowed (%)	8.28	7.16
Disallowed (%)	0.00	0.22

Supplementary Table 3. Primer list

Human-LPCAT3-F	CCGGCGCGCCATGGCGTCCTCAGCG
Human-LPCAT3-R	CCGCGGCCGCTTCCATCTTCTTTAACTTCTCTTTCC
CHICK-LPCAT3-F	GGCGCGCCATGGCTG
CHICK-LPCAT3-R	GCGGCCGCCTCGGC
C-LPCAT3-I59A-F	TCTCCGTCTCGCCGTGTCCATCCTCATGGGTTAC
C-LPCAT3-I59A-R	GATGGACACGGCGAGACGGAGAGCTTGCTCG
C-LPCAT3-Y143A-F	TGGCCGGCGCCTACTTCACTGCCACCG
C-LPCAT3-Y143A-R	CAGTGAAGTAGGCGCCGGCCATCAGATAAGTCAT
C-LPCAT3-Y151A-F	CCGAGCACGCTGACATCAAGTGGACTATGCC
C-LPCAT3-Y151A-R	CTTGATGTCAGCGTGCTCGGTGGCAGTG
C-LPCAT3-M157A-F	CAAGTGGACTGCGCCCCACTGCGTGC
C-LPCAT3-M157A-R	CAGTGGGGCGCAGTCCACTTGATGTCATAGTG
C-LPCAT3-C160A-F	TGCCCCACGCCGTGCTGACCCTCAAG
C-LPCAT3-C160A-R	GTCAGCACGGCGTGGGGCATAGTCCACT
C-LPCAT3-V213A-F	CCTTCATGGCGGGTCCCCAATTCAGC
C-LPCAT3-V213A-R	GGGGACCCGCCATGAAGGCGCCAT
C-LPCAT3-K297A-F	CATCCTCTACGCGTACGTCACTTGCTGGCT
C-LPCAT3-K297A-R	GTGACGTACGCGTAGAGGATGATCTTGCC
C-LPCAT3-Y298A-F	CTCTACAAGGCCGTCACTTGCTGGCTGGTCACC
C-LPCAT3-Y298A-R	CAAGTGACGGCCTTGTAGAGGATGATCTTGCC
C-LPCAT3-F344A-F	CACCCCTTTAGCTACCGGCACCATCGCCAGC
C-LPCAT3-F344A-R	GTGCCGGTAGCTAAAGGGGTGGTCTCGT
C-LPCAT3-N352A-F	GCCAGCTTCGCCATCAATACCAACGCTTGGGTGGCTCGTTAC
C-LPCAT3-N352A-R	TGGTATTGATGGCGAAGCTGGCGATGGTGCCGGTAAATAAAGG
C-LPCAT3-F364A-F	TCGTTACGTCGCCAAGCGCCTCAAGTTTTTAGG
C-LPCAT3-F364A-R	GGCGCTTGGCGACGTAACGAGCCACCCAAG
C-LPCAT3-W387A-F	TTAGCTATCGCGCACGGTTTACATAGCGGTTACCTC
C-LPCAT3-W387A-R	TAAACCGTGCGCGATAGCTAAAAAGAATAAAGCC
C-LPCAT3-H388A-F	GCTATCTGGGCCGGTTTACATAGCGGTTACCTCGTGTGCTTC
C-LPCAT3-H388A-R	CTATGTAAACCGGCCCAGATAGCTAAAAAGAATAAAGCCAGAGCTTG
C-LPCAT3-Y394A-F	CATAGCGGTGCCCTCGTGTGCTTCCAGATGGAG
C-LPCAT3-Y394A-R	CACACGAGGGCACCGCTATGTAAACCGTGC
C-LPCAT3-E401A-F	TTCCAGATGGCGCTGCTGATCGTGATTG
C-LPCAT3-E401A-R	TCAGCAGCGCCATCTGGAAGCACACGAG
C-LPCAT3-I404A-F	GAGCTGCTGGCCGTGATTGTCGAGCGC
C-LPCAT3-I404A-R	GACAATCACGGCCAGCAGCTCCATCTGGAAGC
C-LPCAT3-M446A-F	CTGGATGTTCGCGGGTTATTCTTTAGTGCCTTTCTG
C-LPCAT3-M446A-R	GAATAACCCGCGAACATCCAGTGGTTGGTCTGCT
C-LPCAT3-L450A-F	GGTTATTCTGCAGTGCCTTTCTGTTTATTCACTTGGGA
C-LPCAT3-L450A-R	GAAAGGCACTGCAGAATAACCCATGAACATCCAG
C-LPCAT3-F453A-F	TTAGTGCCTGCCTGTTTATTCACTTGGGACAAGTG

C-LPCAT3-F453A-R	GAATAAACAGGCAGGCACTAAAGAATAACCCATG
C-LPCAT3-T126K-F	GCACCGTGAAAGCCGTGTTTACCACCT
C-LPCAT3-T126K-R	TAAACACGGCTTTCACGGTGCGACCC
C-LPCAT3-Y144K-F	CCGGCTACAAATTCACTGCCACCGAG
C-LPCAT3-Y144K-R	GCAGTGAAGTTTTAGCCGGCCATCAGATAAG
C-LPCAT3-N372K-F	GTTTTTAGGCAAAAAGCTGCTGAGCCAAG
C-LPCAT3-N372K-R	CAGCAGCTTTTTGCCTAAAAACTTGAGGC
C-LPCAT3-H391K-F	GCACGGTTTAAAAAGCGGTTACCTCGTGTG
C-LPCAT3-H391K-R	GTAACCGCTTTTTAAACCGTGCCAGATAGCTAAAAAG
