# Supplementary material for: The structural basis for the phospholipid remodeling by lysophosphatidylcholine acyltransferase 3
Source: Nat Commun. 2021 Nov 25;12:6869. doi: 10.1038/s41467-021-27244-1 (PMC8617236; doi:10.1038/s41467-021-27244-1)
Supplement: Supplementary file 3 — Reporting summary [file 41467_2021_27244_MOESM3_ESM.pdf]

nature portfolio

Corresponding author(s): Yu Cao

Last updated by author(s): Nov 3, 2021

Reporting Summary

Nature Portfolio wishes to improve the reproducibility of the work that we publish. This form provides structure for consistency and transparency in reporting. For further information on Nature Portfolio policies, see our Editorial Policies and the Editorial Policy Checklist.

Statistics

For all statistical analyses, confirm that the following items are present in the figure legend, table legend, main text, or Methods section.

n/a	Confirmed	
☐	☒	The exact sample size (*n*) for each experimental group/condition, given as a discrete number and unit of measurement
☒	☐	A statement on whether measurements were taken from distinct samples or whether the same sample was measured repeatedly
☒	☐	The statistical test(s) used AND whether they are one- or two-sided<br>*Only common tests should be described solely by name; describe more complex techniques in the Methods section.*
☒	☐	A description of all covariates tested
☒	☐	A description of any assumptions or corrections, such as tests of normality and adjustment for multiple comparisons
☐	☒	A full description of the statistical parameters including central tendency (e.g. means) or other basic estimates (e.g. regression coefficient) AND variation (e.g. standard deviation) or associated estimates of uncertainty (e.g. confidence intervals)
☒	☐	For null hypothesis testing, the test statistic (e.g. *F*, *t*, *r*) with confidence intervals, effect sizes, degrees of freedom and *P* value noted<br>*Give P values as exact values whenever suitable.*
☒	☐	For Bayesian analysis, information on the choice of priors and Markov chain Monte Carlo settings
☒	☐	For hierarchical and complex designs, identification of the appropriate level for tests and full reporting of outcomes
☒	☐	Estimates of effect sizes (e.g. Cohen's *d*, Pearson's *r*), indicating how they were calculated

Our web collection on statistics for biologists contains articles on many of the points above.

Software and code

Policy information about availability of computer code

Data collection	The crystals data was collected in Shanghai Synchrotron Radiation Facility (beamline stations BL18U1 and BL19U1). The cryo-EM data was collected by using EPU software (version 2.12.0.2771REL) on Titan Krios, equipment with K3 Summit direct electron detector (Gatan).
Data analysis	tFold (https://drug.ai.tencent.com/console/en/tfold), MotionCorr2-1.1.0, Gctf-v1.18, RELION-3.0, cryoSPARC v3.2.0. T Coot version 0.9.5, BUSTER 2.10.3, Phenix package version 1.19.2, UCSF ChimeraX version 1.2, GraphPad Prism version 6.01.

For manuscripts utilizing custom algorithms or software that are central to the research but not yet described in published literature, software must be made available to editors and reviewers. We strongly encourage code deposition in a community repository (e.g. GitHub). See the Nature Portfolio guidelines for submitting code & software for further information.

Data

Policy information about availability of data

All manuscripts must include a data availability statement. This statement should provide the following information, where applicable:

- Accession codes, unique identifiers, or web links for publicly available datasets
- A description of any restrictions on data availability
- For clinical datasets or third party data, please ensure that the statement adheres to our policy

The PDB number for human LPCAT3 and chicken LPCAT3 were UniProtKB - Q6P1A2 (https://www.uniprot.org/uniprot/Q6P1A2) and UniProtKB - A0A1L1RNG8 (https://www.uniprot.org/uniprot/A0A1L1RNG8), respectively. The coordinates data in this manuscript are deposited at Protein Data Bank with accession codes: 7EWT, 7F3X, and 7F40. The cryo-EM maps have been deposited in the Electron Microscopy Data Bank (EMDB) with accession codes EMD-31442 and -31443.

Field-specific reporting

Please select the one below that is the best fit for your research. If you are not sure, read the appropriate sections before making your selection.

- [x] Life sciences
- [ ] Behavioural & social sciences
- [ ] Ecological, evolutionary & environmental sciences

For a reference copy of the document with all sections, see nature.com/documents/nr-reporting-summary-flat.pdf

Life sciences study design

All studies must disclose on these points even when the disclosure is negative.

Sample size	The structural data for biological macromolecules are validated by physical and chemical laws, instead of statistics. For the enzymatic assay, three times repeat was applied to ensure the all the results all repeatable and reliable.
Data exclusions	For structural and functional experiments shown in the manuscript, all of the data were used.
Replication	For crystallization and cryo-EM sample preparation, more than five times repeat was applied, we always can get valid data for analysis.
Randomization	No randomization is applicable for crystallography to structural biology. For single particle analysis of EM, samples were allocated into experimental groups randomly.
Blinding	Blinding is not necessary or valid for the purposes of structural determination. For functional analysis, blinding was not necessary due to the quantitative nature of the experiment. All experimental data acquired in included in our statistical analysis.

Reporting for specific materials, systems and methods

We require information from authors about some types of materials, experimental systems and methods used in many studies. Here, indicate whether each material, system or method listed is relevant to your study. If you are not sure if a list item applies to your research, read the appropriate section before selecting a response.

Materials & experimental systems

n/a	Involved in the study	
☐	☒	Antibodies
☐	☒	Eukaryotic cell lines
☒	☐	Palaeontology and archaeology
☒	☐	Animals and other organisms
☒	☐	Human research participants
☒	☐	Clinical data
☒	☐	Dual use research of concern

Methods

n/a	Involved in the study	
☒	☐	ChIP-seq
☒	☐	Flow cytometry
☒	☐	MRI-based neuroimaging

Antibodies

Antibodies used	Primary antibodies: Mouse anti Strep II-Tag mAb (ABclonal, AE066); DYKDDDDK Tag Mouse mAb (Cell Signaling, #8146); GAPDH Monoclonal Antibody (proteintech, #60004-1-Ig). The secondary is Anti-mouse IgG, HRP-linked Antibody(Cell Signaling, #7076). All antibodies were diluted by 5% skim milk in TBST (1:10000).
Validation	validation statement available at the web page for each antibodies:<br>Strep II-Tag mAb: https://abclonal.com.cn/catalog/AE066<br>DYKDDK Tag Mouse mAb: https://www.cellsignal.cn/products/primary-antibodies/dykddddk-tag-9a3-mouse-mab-binds-to-same-epitope-as-sigma-s-anti-flag-m2-antibody/8146<br>GADPH Monoclonal Antibody: https://www.ptgcn.com/products/GAPDH-Antibody-60004-1-Ig.htm<br>Anti-mouse IgG: https://www.cellsignal.com/products/secondary-antibodies/anti-mouse-igg-hrp-linked-antibody/7076

Eukaryotic cell lines

Policy information about cell lines

Cell line source(s)	Spodoptera frugiperda cell line Sf9; Expi293 cell line.
Authentication	The cell lines are used to produce proteins for structural determination and analysis. It was purchased and the authentication is not conducted.
Mycoplasma contamination	No mycoplasma contamination was detected.

Commonly misidentified lines<br>(See ICLAC register)	To our best knowledge, there is no commonly misidentified lines.
