# Supplementary figures and images for: The structural basis for the phospholipid remodeling by lysophosphatidylcholine acyltransferase 3
Source: Nat Commun. 2021 Nov 25;12:6869. doi: 10.1038/s41467-021-27244-1 (PMC8617236; doi:10.1038/s41467-021-27244-1)

**Figure 1a**

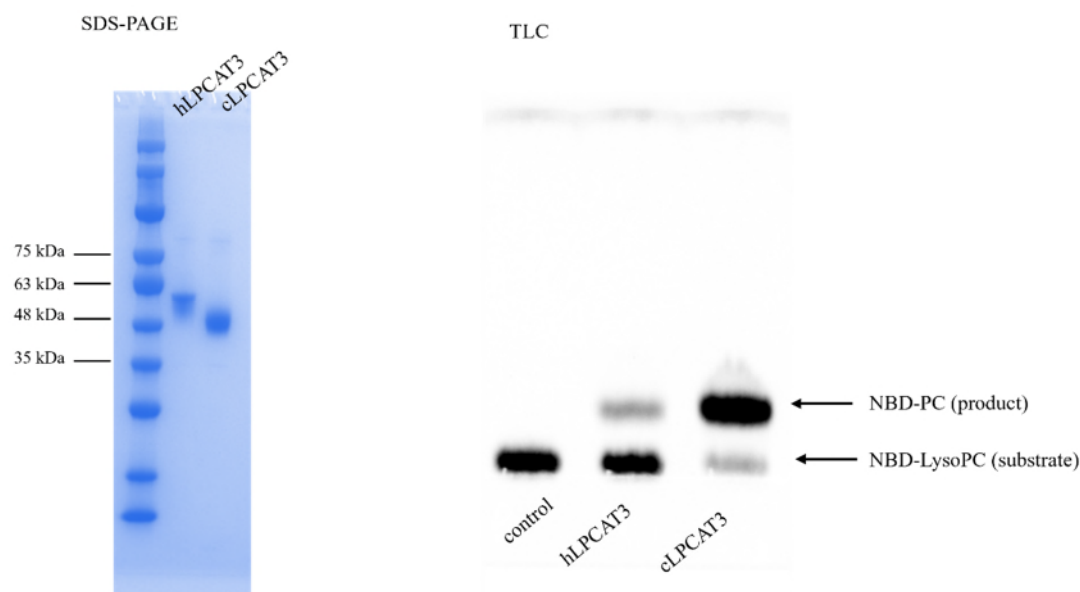

**Figure 6d**

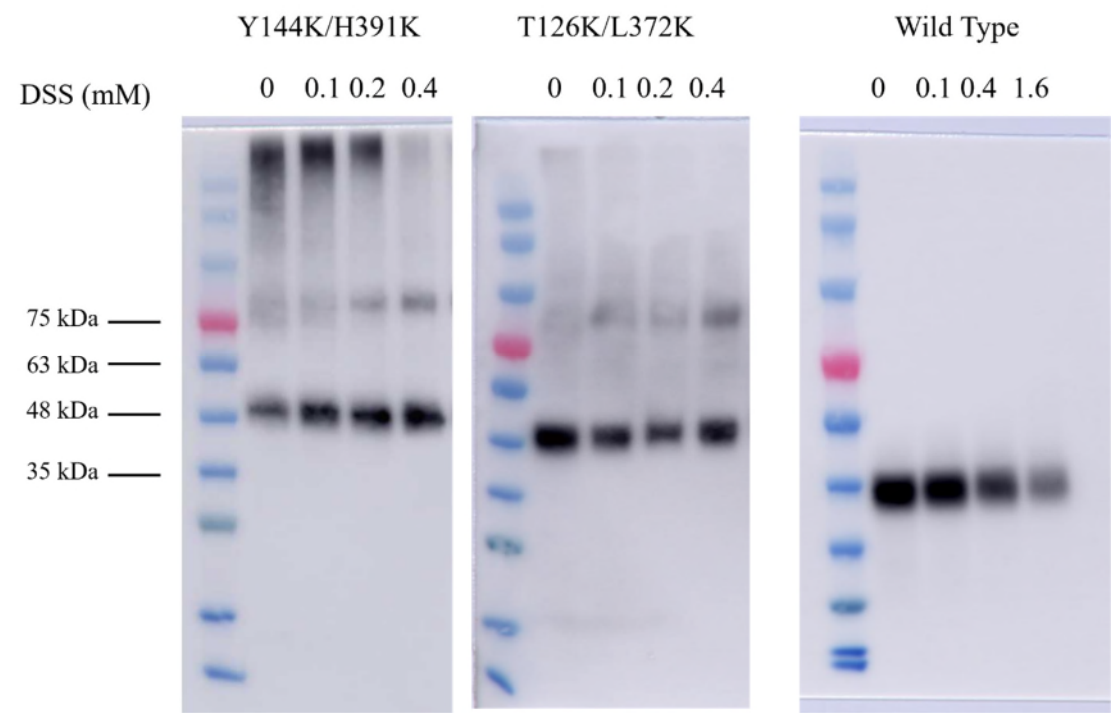

## Supplementary Figure 2b

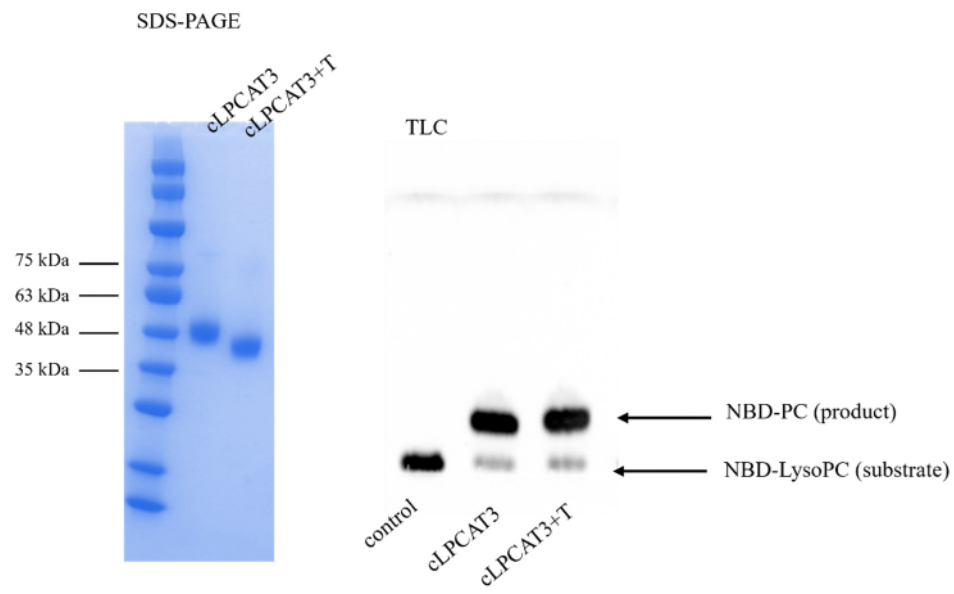

## Supplementary Figure 2c

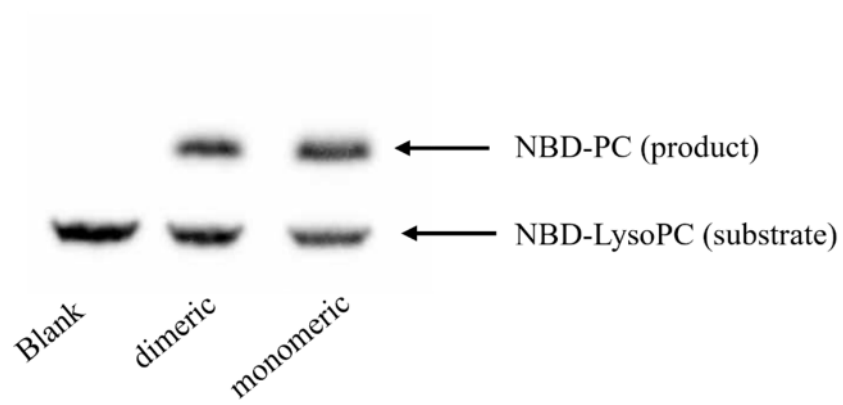

Supplementary Figure 10c

d

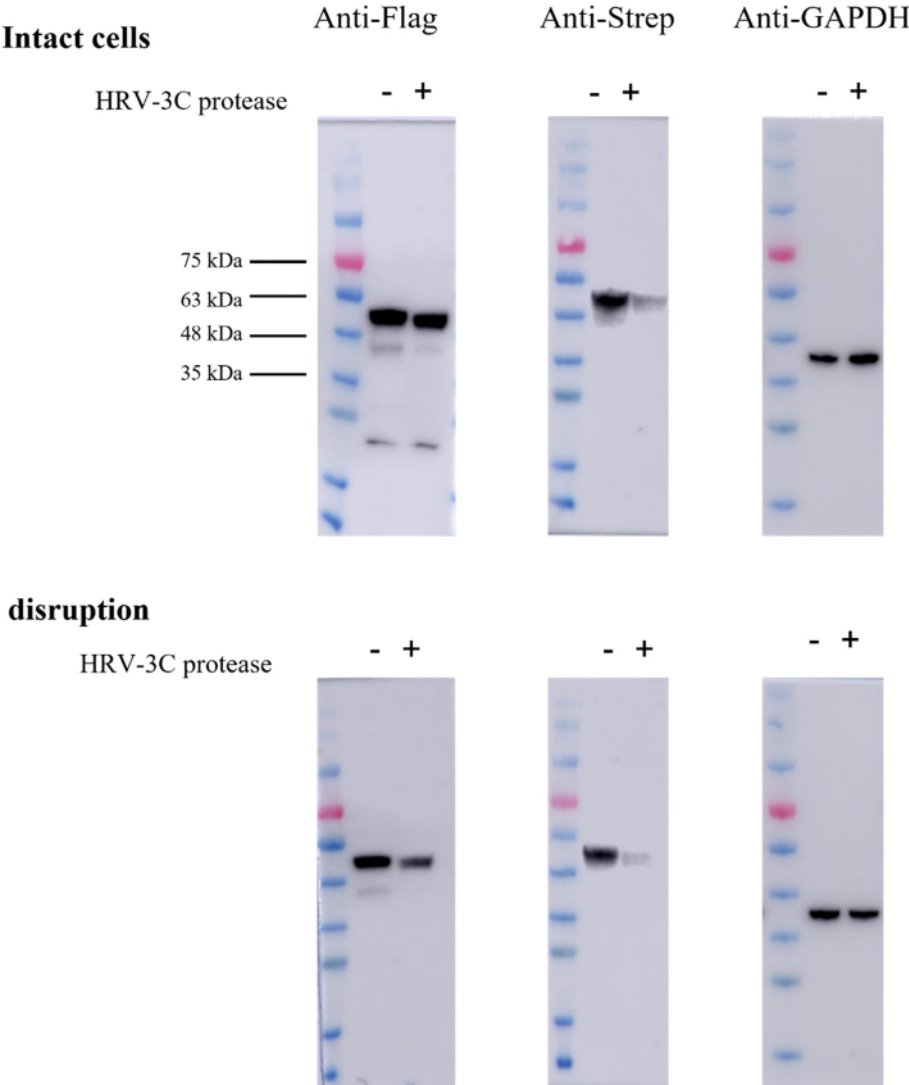

Supplement: Supplementary file 4 — Source Data [file 41467_2021_27244_MOESM4_ESM.zip › Uncropped Gels and Blots.pdf]
